# Evaluation of anatomical pelvic parameters between normal, healthy men and women using three-dimensional computed tomography: a cross-sectional study of sex-specific and age-specific differences

**DOI:** 10.1186/s13018-019-1165-2

**Published:** 2019-05-09

**Authors:** Norio Imai, Hayato Suzuki, Asami Nozaki, Dai Miyasaka, Kazuki Tsuchiya, Tomoyuki Ito, Izumi Minato, Naoto Endo

**Affiliations:** 1 Division of Comprehensive Geriatrics in Community, Niigata University Graduate School of Medical and Dental Sciences, 1-757, Asahimachi-do-ri, Chuou ku, Niigata City, Niigata Prefecture 951-8167 Japan; 2 Division of Orthopedic Surgery, Department of Regenerative and Transplant Medicine, Niigata University Graduate School of Medical and Dental Sciences, Niigata, Japan; 3 Division of Advanced Materials Science and Technology, Niigata University Graduate School of Science and Technology, Niigata, Japan; 4 Division of Orthopedic Surgery, Saiseikai Niigata Daini Hospital, Niigata, Japan; 5 Division of Orthopedic Surgery, Niigata Riko Hospital, Niigata, Japan

**Keywords:** Pelvic incidence, Pelvic tilt, Sacral slope, Three-dimensional measurement, Measurement error, Pelvic morphological parameters, Sagittal spinal balance, Sagittal alignment, 3D bone model, Anatomical parameters

## Abstract

**Background:**

Sagittal spinal balance and standing posture are affected by pelvic morphology, especially pelvic incidence (PI). However, it is not difficult to identify the hip center because of overlap of the pelvis, image contrast, and soft tissue artifacts. Measurements of PI are not always suitable in all patients, especially those with osteoarthritis of the hip joint whose femoral head is nonspherical, subluxed, or dislocated. We measured PI, pelvic tilt (PT), and sacral slope (SS) as anatomical parameters using a novel three-dimensional measurement in order to compare the pelvic morphology between normal, healthy men and women.

**Methods:**

In this cross-sectional study, we evaluated 108 Japanese subjects (55 men, 53 women) without low back or knee pain. We used the three-dimensional pelvis model adjusted to the anterior pelvic plane and measured the pelvic parameters. The subjects were stratified by age (< 50 versus ≥ 50 years) and sex. Intraobserver and interobserver reliabilities were calculated with intraclass correlation coefficients.

**Results:**

There was no significant difference in PI, anatomical-PT, and anatomical-SS between sexes. There was a strong correlation between PI and anatomical-SS in men and women (*R* = 0.790 and 0.715, respectively). Values of anatomical-PT were lower, and values of anatomical-SS were greater among older subjects than among younger subjects; the value of PI was similar between younger and older subjects. Intraobserver and interobserver mean absolute differences were about 2 mm and 2°, respectively; the intraclass correlation coefficient was > 0.87.

**Conclusions:**

We found a strong correlation between PI and anatomical-SS in men and women. This novel measurement concept may be useful to estimate PI from anatomical-SS because the measurements of PI are not always suitable in all patients, especially those with osteoarthritis of the hip joint whose femoral head is not spherical or whose femoral head is subluxed or dislocated. This is the first report to describe the relationship between PI, anatomical-PT, and anatomical-SS as morphologic parameters with a high interclass correlation coefficient for intraobserver and interobserver reliabilities.

## Background

Sagittal spinal balance and standing posture are affected by pelvic morphology, especially pelvic incidence (PI) [1, 2]. Abnormal sagittal alignment of the spine may lead to difficulty in maintaining proper balance, i.e., “hip-spine syndrome” [3]. Sacral slope (SS) and pelvic tilt (PT) [1, 2] are commonly used pelvic parameters; they are considered positional parameters because they are affected by the position of the subject when they are examined with two-dimensional (2D) radiographic images. Conversely, PI is considered an anatomical parameter because it does not vary regardless of the subject’s position [1, 2].

The importance of PI was previously reported for regulating sagittal balance, which leads to optimal lordosis of the lumbar spine and thoracic kyphosis [4]. Moreover, several investigations have reported significant correlations between PI and 2D-PT and 2D-SS [5–8].

Pelvic parameters, even PI, measured by using 2D radiographic images are influenced by the rotation of the pelvis [9, 10]. Many studies have reported the disadvantage of the 2D measurement with regard to accuracy; measurement error was between 3 and 6° [11, 12].

Vrtovec et al. [13] originally described that the three-dimensional (3D) measurement by using reconstructed images from computed tomography are not affected by the projection plane, rotation, and/or lateral tilt of the pelvis; additionally, it is not difficult to identify the hip center because of overlap of the pelvis, image contrast, and soft tissue artifacts [14–16]. Measurements of PI are not always suitable in all patients, especially those with osteoarthritis of the hip joint whose femoral head is nonspherical, subluxed, or dislocated.

Moreover, 2D-PT and 2D-SS change depending on the subject’s position; therefore, the relationship of these angles as anatomical angles is unclear.

If the bone model of the pelvis is adjusted to the same reference plane of the pelvis, PI, PT, and SS are all considered as anatomical parameters. There are no reports on the relationship between PI, anatomical-PT, and anatomical-SS.

The purposes of this study were to evaluate pelvic morphological parameters using a 3D measurement obtained by computed tomography and compare them between normal, healthy men and women and younger and older subjects. We also validated this 3D measurement by using intraobserver and interobserver reliabilities. We hypothesized that the relationship between PI, anatomical-PT, and anatomical-SS would be different between men and women because the morphological feature of the pelvis is different between men and women [17, 18]. This 3D measurement may have a high reliability.

## Methods

### Subjects

For this cross-sectional study performed between August 1, 2010, and December 31, 2010, we recruited 108 healthy Japanese subjects (55 men and 53 women) without lumbago or knee pain and without any abnormal findings of the hip, knee, and spine on radiographic examination.

Computed tomography scans from all participants were examined to reconstruct a 3D bone model [19–21].

This study was approved by the institutional research board of the university, and written informed consent was obtained from all participants. The study population was stratified by age (< 50 vs. ≥ 50 years) and sex (male subjects vs. female subjects).

### Coordinate system of the pelvis

We used ZedHip® software (Lexi, Tokyo, Japan) to create a 3D digital bone model from computed tomography data that accurately reproduced the spatial relationship between the pelvis and femur [19–21]. We adjusted the 3D pelvis model to the anterior pelvic plane [22]. The pelvic *X*-axis (*X*_*p*_ axis), *Y*_*p*_ axis, and *Z*_*p*_ axis were defined according to definitions in previously reported protocols [15, 23].

### Measurements of the pelvic parameters

**Fig. 1:**
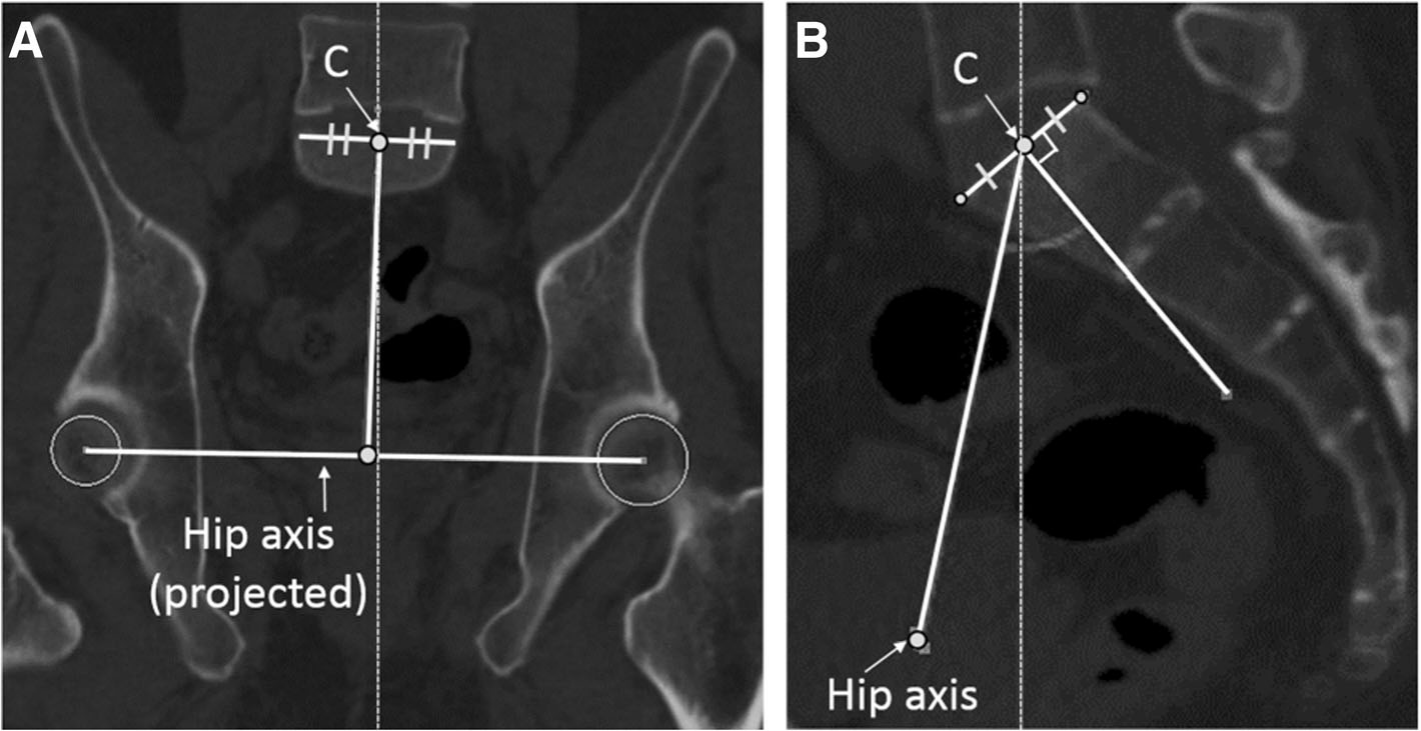
Definition of the center of the sacral end plate of S1. The center of the sacral end plate of S1 (c) is defined as the point that divided the right and left halves in the coronal plane (**a**) and divided the front and rear halves in the sagittal plane (**b**)

**Fig. 2:**
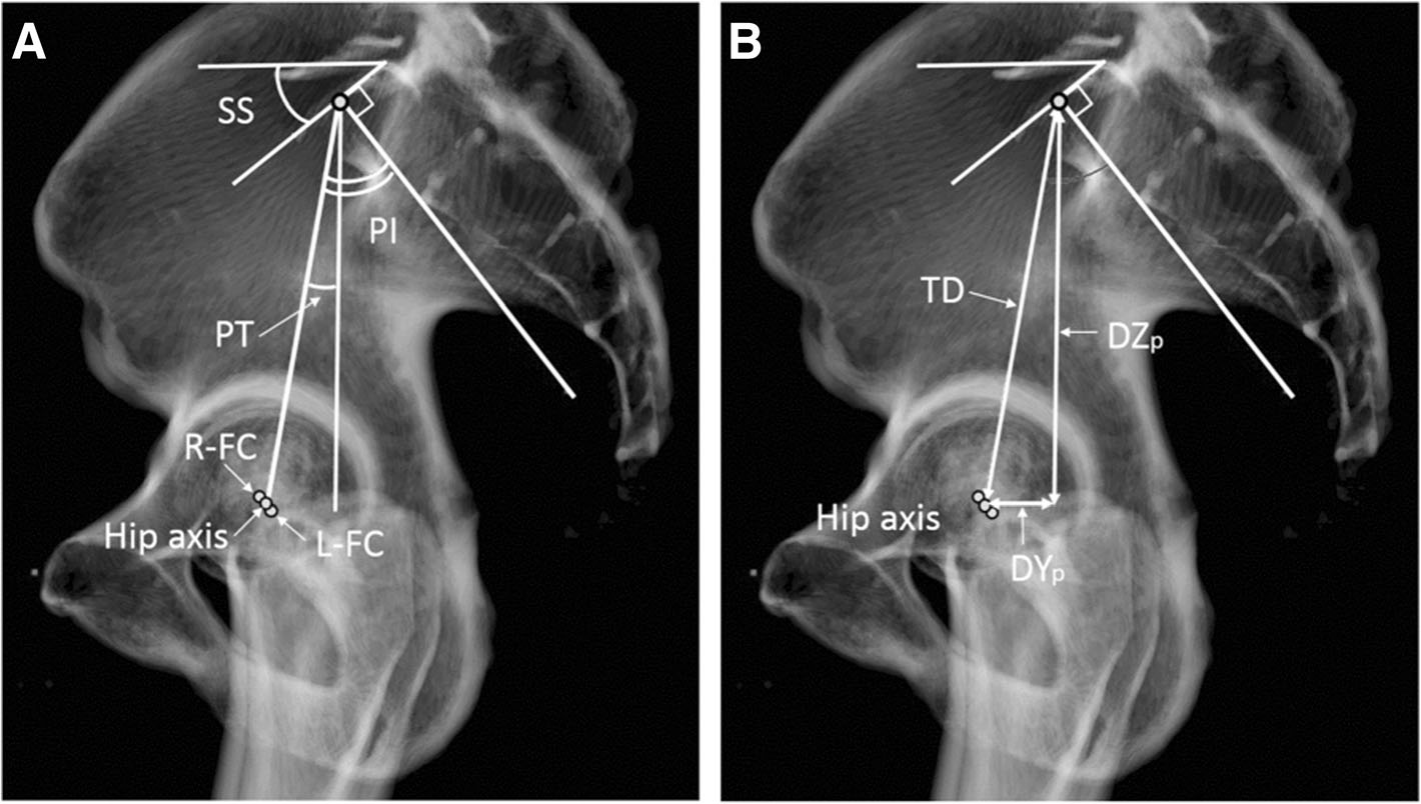
Pelvic parameters in the sagittal plane. PI, PT (anatomical-PT), and SS (anatomical-SS) are considered as anatomical parameters because APP was corrected to 0° in this study (**a**). PI pelvic incidence, anatomical-PT anatomical pelvic tilt, anatomical-SS anatomical sacral slope, R-FC center of the femoral head on the right, L-FC center of the femoral head on the left (**a**), TD total distance between the center of the sacral end plate and hip axis, D*Y*_*p*_ distance of the *Y*_*p*_ coordinate, D*Z*_*p*_ distance of the *Z*_*p*_ coordinate (**b**)

Measurements of the pelvic parameters were performed after the anterior pelvic plane was corrected to 0°, so it was perpendicular to the base plane. Therefore, PI, PT (anatomical-PT), and SS (anatomical-SS) were considered as anatomical parameters in this study. The center of the sacral end plate of S1 (C) was defined as the point that divided the right and left halves in the coronal plane, and divided the front and rear halves in the sagittal plane (Fig. 1a, b). The PI angle was defined between the line perpendicular to the inclination of the superior end plate of S1 and the line connecting the center of the sacral end plate with the hip axis that connected the centers of both femoral heads, projected in the sagittal plane (Fig. 2a). Anatomical-PT was defined as the angle between the line connecting the midpoint of the sacral plate to the hip axis and the vertical line projected in the sagittal plane (Fig. 2a). Anatomical-SS was defined as the angle between the superior end plate of S1 and the horizontal line projected in the sagittal plane (Fig. 2a).

The total distance between the center of the sacral end plate and hip axis projected in the sagittal plane was measured, and distances with regard to each anteroposterior direction, *Y*_*p*_ coordinate of the pelvis (D*Y*_*p*_) and craniocaudal direction *Z*_*p*_ coordinate of the pelvis (D*Z*_*p*_) were measured same as our previous study [16], because total distance, D*Y*_*p*_, and D*Z*_*p*_ potentially affected PI and anatomical-PT (Fig. 2b).

### Statistical analysis

All parameters are reported as average ± 2 standard deviation. We used SPSS statistical software, version 24 (SPSS, Inc., Chicago, IL) to analyze the data. Differences between PI, anatomical-PT, anatomical-SS, and the distance between the center of the sacral end plate and hip axis projected in the sagittal plane in the study groups were analyzed using the paired *t* test. We used Pearson coefficients to determine the correlation coefficients of the pelvic parameters. To evaluate variation, we calculated the mean absolute difference, variability (standard deviation), and intraobserver and interobserver reliabilities with intraclass correlation coefficients and a two-sided 95% confidence interval. We measured 1-week intervals for intraobserver reliability at least twice. A *p* value < 0.05 was considered statistically significant. We compared the measurements of another observer to assess the interobserver reliability. We also performed a post hoc analysis to evaluate statistical power (type II (*β*) error). We defined the effect size (*d*) as 0.5 and type I (*α*) error as 0.05 according to the *t* test, and the effect size (*d*) as 0.3 and type I (*α*) error as 0.05 in the correlation analysis.

## Results

Details of the 108 participants are as follows: 55 men (age 49.3 ± 30.1 [range 19–79] years, body height 166.0 ± 11.4 [range 153–179] cm, body weight 63.3 ± 16.6 [range 42–79] kg, body mass index 22.9 ± 4.5 [range 15.6–29.3] kg/m^2^) and 53 women (age 49.1 ± 29.6 [range 18–79] years, body height 151.9 ± 12.1 [range 135–164] cm, body weight 52.4 ± 14.6 [range 43–73] kg, body mass index 22.7 ± 5.6 [range 17.0–29.6] kg/m^2^). The group of subjects younger than 50 years consisted of 26 men (28.1 ± 21.6 [range 19–49] years) and 28 women (31.2 ± 22.0 [range 18–46] years), whereas the group of subjects aged 50 years or older consisted of 29 men (64.5 ± 20.8 [range 53–79] years) and 25 women (63.8 ± 21.9 [range 53–79] years).

There was no significant difference in PI, anatomical-PT, anatomical-SS, the distance in the *Y*_*p*_ coordinate, distance in the *Z*_*p*_ coordinate, and total distance between men and women (Table 1). There was a strong correlation between PI and anatomical-SS in both men and women (*R* = 0.790 and 0.715, respectively) (Tables 2 and 3).

**Table 1:** Difference in anatomical pelvic parameters between male and female subjects

	Male subjects	Female subjects	*p* value
PI (°)	46.2 ± 19.2 (16.7 to 62.0)	47.0 ± 16.3 (24.7 to 68.9)	0.653
Anatomical-PT (°)	10.3 ± 11.9 (− 4.0 to 21.4)	10.0 ± 14.6 (− 6.6 to 23.5)	0.803
Anatomical-SS (°)	35.9 ± 16.1 (10.6 to 54.7)	37.1 ± 15.5 17.6 to 52.9)	0.472
Total distance (mm)	107.0 ± 19.9 (86.3 to 151.2)	108.4 ± 17.0 (91.2 to 133.2)	0.942
D*Y*_*p*_ (mm)	18.8 ± 21.2 (− 6.9 to 41.1)	18.3 ± 26.1 (− 11.7 to 42.3)	0.836
D*Z*_*p*_ (mm)	104.7 ± 20.5 (80.6 to 151.2)	105.9 ± 18.8 (89.0 to 133.1)	0.539

Upper low: average ± 2 standard deviation, lower low: range

*PI* pelvic incidence, *PT* pelvic tilt, *SS* sacral slope, *DY*_*p*_ distance of the *Y*_*p*_ coordinate, *DZ*_*p*_ distance of the *Z*_*p*_ coordinate

**Table 2:** Correlation between PI, anatomical-PT, and anatomical-SS in male subjects

	Anatomical-PT			Anatomical-SS		
	Total	< 50	≥ 50	Total	< 50	≥ 50
PI (°)	0.543^†^ < 0.001*	0.548^†^ < 0.001*	0.559^†^ < 0.001*	0.790^†^ < 0.001*	0.870^†^ < 0.001*	0.754^†^ < 0.001*
Anatomical-PT (°)				− 0.086^†^ 0.543*	0.064^†^ 0.757*	− 0.075^†^ 0.697*

*PT* pelvic tilt, *SS* sacral slope

^†^Upper low: correlation coefficient

*Lower low: *p* value

**Table 3:** Correlation between PI, anatomical-PT, and anatomical-SS in female subjects

	Anatomical-PT			Anatomical-SS		
	Total	< 50	≥ 50	Total	< 50	≥ 50
PI (°)	0.516^†^ < 0.001*	0.510^†^ < 0.010*	0.494^†^ < 0.010*	0.715^†^ < 0.001*	0.716^†^ < 0.001*	0.676^†^ < 0.010*
Anatomical-PT (°)				− 0.403 0.003	− 0.340 0.112	− 0.392^†^ 0.039*

*PT* pelvic tilt, *SS* sacral slope

^†^Upper low: correlation coefficient

*Lower low: *p* value

With regard to age-specific differences, the values of anatomical-PT were lower (*p* values, men 0.019, women 0.048) and values of anatomical-SS were greater among older men and women (*p* values, men 0.039, women 0.012), whereas the value of PI was similar between younger and older male and female subjects (*p* values, men 0.960, women 0.360) (Tables 4). Moreover, the distance in the *Y*_*p*_ coordinate was significantly lower among older male and female subjects (*p* values, men 0.009, women 0.046), whereas there were no significant differences in the distance in the *Z*_*p*_ coordinate (*p* values, men 0.362, women 0.196) and total distance (*p* value, men 0.173, women 0.128) (Table 4).

**Table 4:** Difference in anatomical pelvic parameters between < 50 and ≧ 50 years in age

	< 50	≥ 50	*p* value
PI (°)			
Male	46.1 ± 20.6 (16.7 to 62.0)	46.3 ± 19.3 (20.9 to 59.7)	0.960
Female	45.8 ± 16.7 (24.7 to 59.3)	47.2 ± 16.0 (35.8 to 68.9)	0.360
Anatomical-PT (°)			
Male	12.2 ± 10.1 (3.5 to 20.9)	8.5 ± 11.9 (− 4.0 to 21.4)	0.019
Female	11.7 ± 13.9 (6.9 to 11.5)	8.5 ± 15.2 (− 3.7 to 23.4)	0.048
Anatomical-SS (°)			
Male	33.9 ± 17.2 (10.6 to 47.0)	37.8 ± 16.1 (19.9 to 54.7)	0.039
Female	34.1 ± 16.3 (17.6 to 48.7)	39.5 ± 13.9 (26.4 to 52.9)	0.012
Total distance (mm)			
Male	108.9 ± 19.1 (86.3 to 123.7)	105.2 ± 19.9 (92.3 to 151.2)	0.173
Female	110.2 ± 17.8 (97.4 to 133.2)	107.0 ± 16.8 (91.2 to 124.7)	0.128
D*Y*_*p*_ (mm)			
Male	22.7 ± 17.6 (7.5–41.1)	15.3 ± 21.2 (− 6.9 to 37.7)	0.009
Female	21.8 ± 24.5 (− 7.1 to 42.3)	15.2 ± 26.6 (− 11.7 to 41.1)	0.046
D*Z*_*p*_ (mm)			
Male	106.1 ± 20.0 (80.6 to 123.5)	103.5 ± 20.6 (90.5 to 151.2)	0.362
Female	107.7 ± 19.8 (93.6 to 133.1)	103.3 ± 19.2 (89.0 to 124.7)	0.196

Upper low: average ± 2 standard deviation, lower low: range

*PI* pelvic incidence, *PT* pelvic tilt, *SS* sacral slope, *DY*_*p*_ distance of the *Y*_*p*_ coordinate, *DZ*_*p*_ distance of the *Z*_*p*_ coordinate

Regarding validation, the results showed that intraobserver mean absolute differences were 1.8 mm for total distance and 1.8° for PI and anatomical-SS, and the minimal correlation coefficient was 0.890 for anatomical-SS (Table 5). However, the interobserver mean absolute difference was slightly larger than the intraobserver mean absolute difference (maximum mean absolute differences were 2.1 mm for total distance and 2.2° for anatomical-SS), and the minimal correlation coefficient was 0.876 for anatomical-SS (Table 5).

**Table 5:** Intraobserver and interobserver reliabilities

	Intraobserver reliability		Interobserver reliability	
	MAD ± 2SD	ICC	MAD ± 2SD	ICC
PI (°)	1.8 ± 3.0	0.946	2.1 ± 3.2	0.923
Anatomical-PT (°)	0.7 ± 0.8	0.963	0.8 ± 0.9	0.952
Anatomical-SS (°)	1.8 ± 2.5	0.890	2.2 ± 3.4	0.876
Total distance (mm)	1.7 ± 1.5	0.965	1.8 ± 1.6	0.958
D*Y*_*p*_ (mm)	0.7 ± 1.2	0.972	0.8 ± 1.5	0.967
D*Z*_*p*_ (mm)	0.8 ± 0.9	0.976	1.0 ± 1.9	0.961

*PI* pelvic incidence, *PT* pelvic tilt, *SS* sacral slope, *DY*_*p*_ distance of the *Y*_*p*_ coordinate, *DZ*_*p*_ distance of the *Z*_*p*_ coordinate, *MAD* mean absolute difference, *SD* standard deviation, *ICC* interclass correlation coefficient

With regard to the post hoc analysis, power values were 0.825 according to the *t* test and 0.945 in the correlation analysis of men and women, 0.572 according to the *t* test and 0.745 in the correlation analysis of men aged < 50 years and men aged ≥ 50 years, and 0.559 according to the *t* test and 0.730 in the correlation analysis of women aged < 50 years and women aged ≥ 50 years, respectively.

## Discussion

In our study, we found that pelvic parameters such as PI, anatomical-PT, and anatomical-SS were similar between men and women. Therefore, the relative location of the center of the S1 superior end plate and hip axis was similar between male and female subjects; it was well known that there are several differences in the morphology and/or contour of the pelvis between men and women [17, 18]. Consequently, pelvic parameters are valid to use without any distinction of sex. Moreover, the distance in the *Y*_*p*_ coordinate, distance in the *Z*_*p*_ coordinate, and total distance were also similar between men and women, whereas body height was approximately 1.1 times larger in men than in women in this study. We preliminarily adjusted the total distance of men to conform with the body height of women; the adjusted total distance of men was calculated as 97.9 using the following formula: 107.0 × (151.9/166.0), and the difference of the total distance between men and women increased over 10 mm. Therefore, these distances were considered as relatively larger in women than in men with consideration of the difference in body height. This morphological difference may be due to the width of the birth canal in women.

There was a strong correlation between PI and anatomical-SS in both male and female subjects and younger and older groups. This novel concept may be useful to estimate PI from anatomical-SS because the measurements of PI are not always suitable in all patients, especially those with osteoarthritis of the hip joint whose femoral head is not spherical or whose femoral head is subluxed or dislocated. Based on our results, the formula could be calculated to estimate PI from anatomical-SS as follows: PI = 0.79 × anatomical-SS + 17.76.

With regard to the values of PI, they were similar between male and female subjects and younger and older subjects. For normal subjects, it is generally accepted that PI increases during childhood and then remains unchanged throughout adolescence and adulthood [6, 24]. Our results were similar to those in previous studies [6, 24]. However, several studies reported that PI was influenced by age and significantly larger in late adulthood [25, 26]. The differences between our study and these studies are unclear. These previous studies and our study were cross-sectional, not longitudinal; therefore, further analysis should be done.

We speculated that the sacrum; the center of the S1 superior end plate was tilted anteriorly and translated anteriorly simultaneously with aging; thus, anatomical-PT was lower and anatomical-SS was larger in older subjects than in younger patients in both sexes.

In 2D measurements, 2D-PT and 2D-SS are considered as positional parameters, and only PI is a morphological parameter. Many previous studies have measured PI using 2D sagittal radiographs in standing position [4, 11, 12, 27]. They reported that the measurement error was between 3 and 6° [11, 12]. Regarding the measurement of PI using the 3D method, few studies have been reported, but Vrtovec et al. [13] reported that mean PI was 47° in 370 normal subjects, mean absolute difference was 2.5 ± 2.3°, and the correlation coefficient was 0.946. We also obtained a lower measurement error using this 3D method than using a previously reported 2D measurement [9, 12]. However, it is difficult to examine the computed tomography scans of all patients for several reasons such as the high cost and radiation exposure; therefore, further investigation is required to validate these novel 3D measurements even in the previously reported 2D method. Our results were considered to have high reliability, similar to those of Vrtovec et al. (Table 1).

The current study has several limitations. First, only a few subjects were enrolled. Second, this study was a cross-sectional study; therefore, the differences between younger and older subjects may not always be true due to long-term changes. Further investigation of the relationship between these parameters may be required to explore the detailed changes of these parameters as they are altered by aging.

## Conclusions

We found no differences in anatomical references, such as PI, anatomical-SS, and anatomical-PT, between men and women. However, there was a strong correlation between PI and anatomical-SS among younger and older subjects in both sexes. This novel measurement concept may be useful to estimate PI from anatomical-SS because the measurements of PI are not always suitable in all patients, especially those with osteoarthritis of the hip joint whose femoral head is not spherical or whose femoral head is subluxed or dislocated.

## Abbreviations

2D: Two-dimensional
3D: Three-dimensional
D*Y*_*p*_: Distance of the *Y*_*p*_ coordinate
D*Z*_*p*_: Distance of the *Z*_*p*_ coordinate
ICC: Interclass correlation coefficient
MAD: Mean absolute difference
PI: Pelvic incidence
PT: Pelvic tilt
SD: Standard deviation
SS: Sacral slope
